# Risk factors associated with precancerous cervical lesion among women screened at Marie Stops Ethiopia, Adama town, Ethiopia 2017: a case control study

**DOI:** 10.1186/s13104-018-3244-6

**Published:** 2018-02-20

**Authors:** Roza Teshome Kassa

**Affiliations:** Department of Nursing and Midwifery, School of Allied Health Sciences, College of Health Sciences, Addis Ababa University, Addis Ababa, Ethiopia

**Keywords:** Precancerous cervical lesion, Case control, VIA, Cervical cancer, Ethiopia

## Abstract

**Objective:**

Although cervical cancer is a preventable disease, it remains a leading cause of death among women in developing countries. In this unmatched case control design, 55 cases and 109 controls were included. The main objective of this study was to assess the risk factors of precancerous cervical lesion in Adama town.

**Results:**

A total of 164 participants were recruited in this study. Of the 109 controls, 64 (61%) and 41 (39%) of cases were using oral contraception. Women who were using oral contraception were two times at risk for developing precancerous cervical lesion than who were not using (COR = 2.059 95% CI 1.006, 4.216; AOR = 2.342). Out of 55 cases, 21 (38.2%) cases had a history STI and out of 109 controls, 24 (22.2%) controls had a history of STI. It was revealed that STI has a significant association for developing of precancerous lesion. Women who had a history of STI were two times at risk of developing precancerous cervical lesion than who had no (COR = 2.187; AOR = 2.485). It was found that initiation of sexual intercourse before the age of 15 years has 5.6 risks to develop precancerous cervical lesion (COR = 5.625 AOR = 6.703).

**Electronic supplementary material:**

The online version of this article (10.1186/s13104-018-3244-6) contains supplementary material, which is available to authorized users.

## Introduction

Cervical cancer is a disease in which the cells of the cervix become abnormal and start to grow uncontrollably, forming tumors [1]. Developing countries contribute more than three-quarter of women death in cervical cancer every year and Ethiopia contributes substantially to this figure due to inadequate health care and virtually nonexistent screening mechanisms for early detection [2]. According to 2008 estimates, invasive cervical cancer (ICC) is the second most common cancer in women worldwide. Although cervical cancer is a preventable disease, it remains a leading cause of death among women in developing countries. In 2012 there were an estimated 528,000 new cases of cervical cancer and 266,000 deaths from cervical cancer, with 70% of those deaths occurring in developing countries [3].

In Africa, according to most recent estimates, 80,400 women are diagnosed with cervical cancer every year, the second most frequent cancer. 50,300 die from the disease every year, the leading cause of cancer death. In sub-Saharan Africa, cervical cancer accounts for 22.5% of all cancer cases in women, and the majority of women who develop cervical cancer live in rural areas [3, 4].

Of nearly 22 million Ethiopian women over the age of 15, approximately 7600 are diagnosed with cervical cancer and roughly 6000 women die of the disease each year. On the other hand, the incidence and mortality from cervical cancer in Ethiopia is 26.4 and 18.4/100,000 respectively. These figures are probably lower than the actual number of cases, given the low level of awareness, cost, and limited access to screening services and lack of a national cancer registry [4, 5].

Human papilloma virus (HPV) is the primary etiologic agent of cervical cancer. It is the most common viral infection of the reproductive tract. Most sexually active women and men will be infected at some point in their lives and some may be repeatedly infected. The peak time for acquiring infection for both women and men is shortly after becoming sexually active. It is responsible for 99% of cervical cancer and accounts for approximately half of the infection related burden of cancer in women. There are over 100 types of HPV. The genital-type HPVs are divided into high, intermediate and low-risk types according to their association with genital tract cancer. High risk types of HPV (HPV-16, -18, -31, -45) account for more than 90% of cervical carcinoma [6].

Several screening methods to detect precancerous and cancer are available, and can be performed safely and inexpensively in an outpatient setting. In many developing countries, treatment of precancerous is neglected because therapeutic services are unavailable, inaccessible, or inadequately linked to screening services.

The high burden of cervical cancer combined with the lack of infrastructure and financial resources for cytology based screening programs has led to the search for alternative strategies for cervical cancer prevention in low-resource settings [7]. The most widely implemented low-cost screening technique is visual inspection with 3–5% acetic acid (VIA) [8].

## Main text

### Methods

The study was conducted in Adama town, Marie Stops International Hospital. An institutional based retrospective case control design was conducted. The sample size of this unmatched case–control study was calculated by using Stat cal in epi-info 7 by the following assumptions. Level of confidence 95, 80% power, proportion of controls who begun sexual intercourse before the age of 20 year odds ratio of 60% for the development of precancerous lesions of the cervix [17], Odds ratio 3 and case to control ratio 1:2. This gives the total sample size of 164 individuals: 55 cases and 109 controls.

Structured data abstraction form was developed after reviewing the client record/log book and literatures. Fifty-five cases were identified by registration identification number and included in the study. The next two controls were included consecutively. For any missed data of control the next serial number was included. Ethical approval was obtained from the ethical review board of the Rift Valley University. Client records were treated confidentially and name of clients was not included in the data collection. Data was entered, cleaned and analyzed by SPSS version 20 statistical package. Descriptive summaries using frequencies and proportions were used to present the study results. Binary and multivariable logistic regressions were used to identify factors associated with the pre-cancerous cervical lesion. Adjusted odds ratio at 95% confidence interval and P-value were used to measure the strength of association and identify statistical significant result. P-value < 0.05 was considered as a statistically significant association.

### Results

#### Socio demographic characteristics

One hundred and sixty-four participants were recruited in this study. From these, 109 were controls who were VIA negative and 55 were cases who were VIA positive. The mean age was 35.7 years. Most of the controls 104 (68.9%) were married and 47 (31.7%) of cases were married. Of control groups, 14 (12.8%) and 3 (5.5%) of cases were uneducated (Table 1).

**Table 1:** Socio demographic characteristics, case control study, Marie Stops Ethiopia, Adama, 2017

Variables	Cases (%)	Controls (%)
Age (years)		
30–40	48 (87.2)	96 (88.1)
40–60	6 (10.9)	11 (10.1)
> 60		1
Level of education		
Elementary	12 (21.8)	25 (22.9)
High school	30 (54.5)	37 (33.9)
College	10 (18.2)	33 (30.3)
None	3 (5.5)	14 (12.8)
Marital status		
Married	53 (96.4)	101 (92.7)
Divorced	2 (3.6)	6 (5.5)
Widowed	0	1
Single	0	1

#### Reproductive health history

Among the control group, 105 (96.3%) and 53 (96.4%) of cases had children. Of these, 96 (91.4%) of controls and 50 (94.3%) of cases had less than four children while 9 (8.6%) of controls and 3 (5.7%) cases had more than 4 children. More than half of the controls, 64 (58.7%) and 41 (74.5%) of cases were using contraception. From the control group, 24 (22%) and 21 (38.2%) of cases had a history STIs (Table 2).

**Table 2:** Reproductive health history of study participants, Marie Stops Ethiopia, Adama, 2017

Variables	Cases (%)	Controls (%)
Current use of contraception	41 (39)	64 (61)
History of STI	21 (46.7)	24 (53.3)
Number of children		
< 4 children	50 (34.2)	96 (65.8)
> 4 children	3 (25)	9 (75)
Age of first sexual intercourse (years)		
< 15	20 (55.6)	25 (44.4)
16–20	23 (29.1)	56 (70.9)
21–25	6 (18.2)	27 (81.8)
> 26	1 (14.3)	6 (85.7)
No of sexual partner in life time		
One	15 (18.5)	66 (81.5)
2–5	8 (28.6)	20 (71.4)
> 5	32 (58.2)	23 (41.8)
HIV/AIDS tested		
Yes	42 (33.9)	82 (66.1)
No	12 (32.9)	27 (67.5)
HIV/AIDS result		
Positive	9 (50%)	9 (50)
Negative	33 (30.8)	74 (69.2)
On art		
Yes	5 (50)	5 (50)
No	4 (50)	4 (50)

Among total study subjects 45 (27%) had first sexual intercourse before the age of 15 years. Out of total participants, 55 (33.5%) had more than five sexual partners in their lifetime. The majority of participants, 124 (75.6%) were tested for HIV/AIDS. Of these, 107 (65.2%) of them were HIV negative 18 (11%) of them were HIV positive (Table 2).

#### Risk factors associated with precancerous cervical lesion

Of the 55 cases, 41 (74.5%) of them had been using oral contraceptives. Out of 109 controls, 64 (58.7%) of them had been using oral contraception. It was found that there is a significant association between using oral contraception and developing precancerous cervical lesion. Women who were using oral contraception were two times at risk for developing of precancerous cervical lesion than who were not using (COR = 2.059; 95% CI 1.006, 4.216; AOR = 2.342; P < 0.025) (Table 3).

**Table 3:** Logistic regression analysis of risk factors associated with precancerous cervical lesion

Variables	Cases, n (%)	Controls, n (%)	COR (95% CI)	AOR (95% CI)	P-value
Use of oral contraception					
No	14 (25.5)	45 (41.3)	1	1	0.048
Yes	41 (74.5)	64 (58.7)	2.059 (1.006, 4.216)	2.342 (1.114, 4.923)	
History of STI					
No	34 (61.8)	85 (77.9)	1	1	0.030
Yes	21 (38.2)	24 (22.1)	2.187 (1.078, 4.440)	2.485 (1.192, 5.180)	
No of children					
< 4 children	50 (90.9)	96 (88.1)	1	1	0.517
> 4 Children	5 (9.1)	3 (2.8)	0.640 (0.166, 2.470)	0.481 (0.118, 1.955)	
Age of first intercourse (years)					
< 15	25 (45.5)	20 (18.3)	5.625 (1.9245, 16.271)	6.703 (1.735, 10.123)	0.001
16–20	19 (34.5)	56 (51.4)	1.214 (0.506, 2.900)	1.253 (0.518, 3.023)	
21–25	10 (18.8)	27 (24.7)	1	1	
> 26	1 (1.8)	6 (5.5)	0.333 (0.035, 3.159)	0.381 (0.034, 3.67)	
No of sexual partner in life time					
1	15 (27.3)	66 (60.6)	1	1	0.000
2–5	8 (14.5)	20 (18.3)	1.760 (0.652, 4.72)	1.480 (0.533, 4.113)	
> 5	32 (58.2)	23 (21.1)	6.121 (2.818, 13.294)	5.864 (2.67, 12.843)	
HIV/AIDS result					
Negative	33 (60)	74 (67.9)	1	1	0.117
Positive	9 (16.4)	9 (8.3)	2.242 (0.816, 6.163)	2.242 (0.816, 6.163)	
Unknown	13 (23.6)	26 (23.9)	1.121 (0.513, 2.451)	1.212 (0.523, 2.452)	
On HAART					
No	4 (44.4)	4 (44.4)	1	1	0.541
Yes	5 (55.6)	5 (56.6)	1.100 (0.156, 6.420)	1.284 (0.158, 10.448)	

Out of 55 cases, 21 (38.2%) cases had a history STI and out of 109 controls, 24 (22.2%) controls had a history of STI. It was revealed that STI has a significant association for developing of precancerous lesion. Women who had a history of STI were two times at risk of developing precancerous cervical lesion than who had no history of STI (COR = 2.187; 95% CI 1.078, 4.440; AOR = 2.485; P < 0.015) (Table 3).

Of the 55 cases 53 of them had children and 3 (5.7%) had more than 4 children. Out of 109 controls, 105 of them had children and 9 (8.6%) of them had more 4 children. It was found that there was no significant association between increasing number of children and developing precancerous cervical lesion after even adjusting other confounding factors (COR = 0.643; 95% CI 0.166, 2.470; AOR = 0.455; P < 0.280) (Table 3).

Out of 55 cases 25 (45.5%) cases, had first sexual intercourse at the age of less than 15 years and out of 109 controls 20 (18.3%) had first intercourse at the age of less than 15 years. It was found that initiation of sexual intercourse before the age of 15 years has 5.6 risks to develop precancerous cervical lesion (COR = 5.625; 95% CI 1.9245, 16.271; AOR = 6.703; P < 0.001) (Table 3).

Among 109 controls 23 (21.1%) had more than five sexual partners in their lifetime, whereas from 55 cases 32 (58.2%) of them had more than five sexual partners in their lifetime. It was revealed that having more five sexual partners has six times risk to develop precancerous cervical lesion (COR = 6.121; 95% CI 2.818, 13.294; AOR = 5.864; 95% CI 2.677, 12.843; P < 0.00) (Table 3).

### Discussion

This study showed that use of oral contraception increases precancerous cervical lesion by twofolds (COR = 2.059; 95% CI 1.006, 4.216; AOR = 2.342; P < 0.025). The possible association between the use of OCP and the development of cervical neoplasia has been the subject of many epidemiological investigations, but the nature of the association remains unclear [9]. In 1993 a massive worldwide study conducted by the World Health Organization, was published which examined the risk established between OCP use and invasive squamous cervical carcinoma among 2300 women who had cervical carcinoma and found a strong correlation [10]. In a large study group, Herrero et al. showed that women who had received injectable progestins for at least 5 years and who had used them at least 5 years ago suffered a 430% increased risk of developing cervical cancer [11]. Also, Briton revealed that usage of OCP for more than 10 years could increase the risk of cervical cancer [12].

In this study, initiation of sexual intercourse earlier than the age of 15 years has 5.6 times risk for the development of precancerous cervical lesion. Similar finding was observed in a study conducted in Jimma, Ethiopia that showed clients who started intercourse less than 16 years were 2.2 times more likely to have a precancerous cervical lesion (AOR [95% CI] 2.2 [1.1, 4.3]) [13]. This association was also seen in a case–control study of cervical cancer risk factors in Indian women where the maximum risk of cervical cancer was increased in women with a sexual debut at < 12 years old (OR = 3.5) and also increased in women who had extramarital sexual relationships (OR = 5.5) [14]. This may be due to Women who had sexual intercourse for the first time at a younger age may have been exposed to a persistent HPV infection for longer periods compared to women who began to have sex at a later age [15].

In the present study there wasa strong association between sexual transmitted infections with precancerous cervical lesion (COR = 2.187; 95% CI 1.078, 4.440; AOR = 2.485; P < 0.015). Similarly, in a study conducted in Aksum, Ethiopia there was a strong association between STI and the development of cervical cancer [AOR = 49.88 (95% CI 16.59, 149.91)] [16].

This study found that having multiple sexual partner and precancerous cervical lesion has a strong association. This was a comparable finding with a study conducted in Rwanda that found multiple sexual partner is a risk factor for cervical cancer (COR = 1.40; 95% CI 0.85–2.30) [17]. This association of cervical cancer with sexual behavior also was shown in a case–control study done in Manchester, England where the number of sexual partners was the risk factor (OR for six or more = 3.89) [18]. This may be due to; sexual activity may increase the chance of transmission of the human papilloma virus (HPV). The probability of encountering an infected partner increases as the number of partners someone has increased. On the other hand, having fewer sexual partners means there are simply fewer chances to come into contact with someone who has HPV infection.

### Conclusion

The majority of women who develop a precancerous cervical lesion tend to have one or more identifiable factor that increases the risk of the disease. Use of current oral contraception has 2 times risk for precancerous cervical lesions. Being infected with STIs, first intercourse at an early age and multiple sexual partners in life time are also independent predictors of precancerous cervical lesions. So that it should be focused on addressing HPV vaccination for girls, avoiding early marriage, promoting use of condom and discouraging initiation of early sexual intercourse (Additional file 1).

## Limitation

- Being a retrospective record review design was the limitation of this study. Data collectors faced challenges in skipping incomplete recorded client cards.

## Additional file

**Additional file 1.** Risk factors of precancerous cervical lesion file.

## Abbreviations

CIN: cervical intraepithelial lesion
LSIL, HSIL: low grade and high grade squamous intraepithelial lesion
HPV: human papilloma virus
HIV: human immunodeficiency virus
ICC: invasive cervical cancer
WHO: World Health Organization
HAART: highly active antiretroviral therapy
VIAC: visual inspection of the cervix with acetic and cervicography
LEEP: loop electrosurgical excision procedure
ART: antiretroviral therapy
OICs: opportunistic infectious conditions clinics
ZDHS: Zimbabwe Demographic Health Survey
